# Physiology

**Published:** 1859-07

**Authors:** S. Weir Mitchell

**Affiliations:** Lecturer on Physiology in the Phila. Med. Association


### PHYSIOLOGY.

BY S. WEIR MITCHELL, M.D.,

LECTURER ON PHYSIOLOGY IN THE PHILA. MED. ASSOCIATION.

### I.—*Rhythmical Contractions of a Voluntary Muscle, the Peroneus Brevis, of the right side.* By JOBERT, (de Lamballe.)

This curious case, reported by Jobert, (de Lamballe,) has considerable interest, as illustrating the mechanism of "knockings," and as showing that a voluntary muscle may exhibit, pathologically, a rythmical contraction so regular as to resemble in this respect the heart-pulse itself. The disease was of six years duration, and was characterized by blows ("battements") which were heard behind the right external malleolus. On a level with the malleolus, and at its posterior border, a regular pulsation could be felt, accompanied by a momentary lifting up of the soft parts, and followed by a dry sound at the close of each muscular contraction. This sound could be heard at some distance, and at all hours, whether the patient was on foot, in or out of bed, awake or asleep. After describing the mechanism of these pulsations, and alluding to the "rappings," M. Jobert remarks that this case shows how, under the influence of muscular contractions, the displaced tendons can produce sounds, at the moment they fall back into their bony channels, which would be, for some persons, "spiritual knockings."

The author demonstrates also that, by practice, any man can acquire this accomplishment, and he recalls the ingenious experiments of M. Schiff, which are already known to most of our readers. M. Schiff considered that all of these sounds were caused by the tendon of the peroneus longus, and that in such persons as possessed the power of "rapping" there was either a weakening or a complete absence of the common sheath of the long and short peronei muscles. M. Jobert admitted the return of the tendon upon the bony gutter as the cause of the sound, but did not think it necessary to presuppose any anomaly in regard to the sheath. M. Velpeau remarked, that these sounds could be produced in many regions, as the hip, the shoulder, or the inner side of the foot. The tendon of the long portion of the biceps muscle produced the sound in question with facility whenever the fibrous bridles which usually retained it in place were relaxed or broken.

M. Jules Cloquet recalled the case of a young girl whose father intended to exhibit her in public, announcing that she had a clock in her stomach. By a slight movement of rotation in the lumbar region of the spine, she produced strong crackling sounds, more or less regular. These noises were audible at the distance of twenty-five feet, and resembled the sound of an old turn-spit or jack.

M. Jobert saw little analogy between this case, where the sounds were produced at will, and that of his own patient, in whom they were regular and involuntary. At the same time he stated, that the situation of the peroneal tendons is so favorable to the production and voluntary control of these sounds, that persons have, by long practice, been thus enabled to execute melodious airs, as the Marseillaise, etc. etc. with perfect regularity, and by the sole aid of the clacking noise caused by displacing and restoring the peroneal tendons.—(*Gazette Hebdom. de Med. et de Chirurgie*, vol. vi. No. 17.)

II.—*Cause of the Tendency of Red-blood Corpuscles to arrange themselves in Piles.* By C. ROBIN

In an interesting paper upon the anatomy of the blood corpuscles, M. Robin thus speaks of the numulated arrangement so often noticed in recently drawn blood:—"This fact has in it nothing which allies it to the vital properties of the anatomical elements in question. It is only a consequence of a commencement of alteration in the blood globules placed outside of the vessels and in a serum which is slightly modified." M. Robin then affirms, that whenever the phenomenon of coherence is seen it is due to a viscous and tenacious transparent matter, which is exuded from the globules, and holds them together when the currents in a drop or in a bowl of blood bring them into contact face to face. To prove the existence of this tenacious layer, M. Robin relies upon these facts:—When the piles of corpuscles are formed as usual, it is possible to break them up by pressing upon the cover glass, when they will separate and at last reunite to other corpuscles, so as to make new groups. When this manœuvre is made with moderate force, it will be seen that there is some difficulty in completing the separation. The globules glide on one another, until they are attached only at the edge, the two globules elongating under the pressure employed. When at length the separation is complete, so that a distinct space exists between the two globules, they will reapply themselves one to the other, as before, if the pressure ceases. It is evident, therefore, that while thus to appearance separated the globules are bound together by a perfectly transparent, but glutinous matter. In some lights and by proper management, it is even possible to see the viscous substance in question. M. Robin thinks that this viscous exudation is due to a slight concentration of the serum in which float the globules.—(*Jour. de Phys.*, April, 1858.)

III.—*Relative Temperature of the Submaxillary Saliva and that of the Blood of the Carotid of the same side.* By C. LUDWIG and A. SPIESS.

By the use of a thermo-multiplier, MM. Ludwig and Spiess have found that if the nerves of the submaxillary gland are galvanized, the temperature of saliva which escapes from its duct is 1·8° F. above that of the carotid arterial blood of the same side. Connecting this with the recent discoveries of Bernard, we perceive that the secreting action of this small gland causes a notable rise in temperature. At the same time there is a diminution in the formation of carbonic acid, as is shown by the fact that during this over-activity of secretion the venous blood of the gland remains red and almost as much charged with oxygen as arterial blood,—another fact to be added to those which already show how insufficient is the theory which attempts to account for animal heat by simple combustion.—(*Jour. de Phys.*, Oct., 1858; *fr. Zeitschrift für Rationellen. Med.*, vol. ii. part ii., 1858, p. 360.)

IV.—*On the Influence of Exercise over Respiration and Pulsation.* By EDWARD SMITH, M.D., etc.

The author has been engaged in a very elaborate examination of the points above mentioned, as well as in regard to the quantity of air inspired under varied circumstances. The instrument employed to measure the air inspired was a very small "dry gas meter," arranged to record 2,000,000 of cubic inches. To the exit pipe of the instrument was adapted a caoutchouc tube, ending in an naso-oral mouth-piece, and so arranged that the air of inspiration passed through the meter and was there recorded, while the expired air passed out through the side of the mouth-piece.

Having ascertained that when seated he inspired a little over 500 cubic inches per minute, he inquired into the change produced by passive and active movements. Thus rolling of the body, after Marshall Hall's plan, without self-exertion, increased the amount inspired 56 cubic inches per minute, and 73 cubic inches more when lying on the face. Swimming in water at 68° F., raised the inspired air to 2200 cubic inches per minute, the respiration to 23, and the pulse to 115. Bending the trunk forward when seated, decreased the inspired air 50 cubic inches per minute. This observation has many interesting hygienic bearings. The passive exercise of riding or driving increased the air inspired in proportion to the roughness of the movement, being largest in equitation and least in easy railway riding.

Active exercise largely increased the amount inspired. Walking raised the amount inspired, so that slow walking elevated it to 1000 cubic inches, and fast walking to 2000 or 2500 cubic inches per minute—the rate of speed remaining the same. The carriage of burdens greatly increased the demand for air.

It is unnecessary to follow the author through the whole of his interesting observations. It is sufficient to add, that the exercise of the treadmill, and of rapid running, gave the maximum numbers, and showed that it is possible that the quantity of air we breathe may be increased sevenfold during short periods. The remarks which follow are too extended for quotation, but will well repay full perusal. They consist in applications of the knowledge acquired by these researches to questions of hygiene, disease, and prison discipline.—(*Edinburgh Medical Journal*, January, 1859.)

V.—*Of Glucogenetic Material, regarded as a Condition of Development of certain Tissues in the Fœtus before the appearance of the Sugar-making Function in the Liver.* By CL. BERNARD, April 4, 1859.

In a recent paper—reported on *page* 533, May, 1859, of this journal—is a statement of the fact announced by M. Bernard, that the placenta in some animals, and the amnios in others, contain a glucogenous compound. Following up the subject, he sought to determine in what other fœtal organs or appendages it also existed, and in what position and form it was to be looked for in these localities.

1. Cutaneous surface.—The glucogenous matter is found infiltrating the tissue of the skin, and within its epithelial cells. When the surface of a young fœtus is scraped the matter so removed consists of various histological elements abounding within and without in a granular matter, which the acidulated tincture of iodine colors wine-red. The glucogenous substance thus identified gradually disappears as development progresses.

2. The mucous membranes.—The epithelia of these surfaces and of the conduits of their glands also yield the same substance, while the tissue proper of the glands annexed to the intestinal canal is devoid of it altogether.

3. In the lung surfaces, it persisted to the period of birth; and it was also found, although not up to this epoch, in and on the mucous surfaces of the genito-urinary passages.

4. Interior tissues.—The granular glucogenous matter is never found in the nerves or bones of the fœtus. Very early in embryonic life it is also absent from the muscles, but a little later it appears in these, and remains a constant element of their bulk until after birth. The glands are entirely free from this ingredient, and it is absent even from the liver until the middle of fœtal life, when that organ assumes its usual glucogenic function, and the glucogenic matter begins to disappear from the other tissues of the economy.

M. Bernard thence concludes that glucogene fulfils an important function in the processes of fœtal development, while, in like manner, the glucogenetic function of the liver in post embryonic life is equally concerned in the phenomena of growth and nutrition.—(*Gazette Hebdom. de Med. et de Chirurgie*, vol. vi. No. 16.)

### VI.—*Physiology of Secretion. Supposed Influence of one Nerve upon another.* By CL. BERNARD.

It is scarcely necessary to state in full the views of M. Bernard in regard to the effect produced by exciting the two sets of nerves which enter the submaxillary gland, in order to make the following additional views comprehensible. When the filaments which emerge from the superior cervical ganglion were excited, the saliva ceased to flow from the gland, its venous blood became very dark, and its circulation was notably diminished. When, on the other hand, the chorda tympani-nerve was irritated, the local circulation became rapid, the emergent blood was red, and passed out in jets, and the secretion of saliva became suddenly active. These facts seemed to accord with the idea of an active dilatation in the vessels; but this view being unsatisfactory to M. Bernard, he sought further for an explanation of a less dubious nature.

Last March, he related to the Society of Biology certain facts which have afforded the foundation of an explanation, which he will seek to test still further by new and varied experiments.

1. When the main cervical cord of the sympathetic nerve is divided below the superior cervical ganglion, no effect is produced upon the circulation of the blood in the submaxillary gland. If, on the other hand, the section involves the nerve branches which pass from the ganglion to the gland, the local circulation becomes thereby increased in a greater or less degree, according as the section is made near to or remote from the gland. This acceleration of the circulation is due to such a paralysis of the vessels as occurs in the face, when the cervical trunk of the sympathetic is divided. If the section of the sympathetic filaments of the submaxillary gland be made in the hilus of that body, the effects alluded to are most marked.

2. When a dog is poisoned with woorara, the above described phenomena—increased circulation, salivation, altered color of the blood—occurs at the moment when the nerves become paralyzed by the action of the poison.

3. Finally, it is possible to poison locally the submaxillary gland, by injecting a minute amount of woorara into its substance, when the same phenomena are observed.

It is thus rendered probable that paralysis of the sympathetic nerve and excitation of the chorda tympani produce like results as regards circulation, secretion, and color of the blood issuing from the submaxillary gland. We should thus be led, following M. Bernard, to consider the sympathetic as governing the vessels, contracting their calibre at times, and at others allowing them to dilate. To explain, however, the action of the chorda tympani upon the circulation of the salivary gland in question, M. Bernard announces the following very singular hypothesis. He supposes the chorda tympani-nerve to act not on the muscle of the vessels, but upon the minute filaments of the sympathetic nerve itself. Being excited, it acts on the sympathetic to cause abolition of its power to contract the size of the vessels. This would be, of course, one nerve acting upon another, the paralysis of one nerve by the excitation of another. It is not difficult to conceive of many possible applications of this view, if it can be shown to be tenable in the case now before us.—(*Gaz. Hebd.*, February, 1859, vol. vi. No. 6.)

### VII.—*Effects of Ligature of the Œsophagus in Animals.* Report of M. TROUSSEAU.

The question here proposed assumes a vast importance, from the fact that in a large number of the experiments upon the ingestion of poisons, made by Orfila and others, the œsophagus was tied to prevent the rejection of the substance employed. Until a recent period, no one seems to have suspected that the mere ligation of the œsophagus could give rise to formidable symptoms and to fatal results, which might be wrongly referred to poisons previously placed in the stomach. In June, 1856, MM. Bouley and Reynal sent to the Academy of Medicine a communication, in which they believed themselves to have demonstrated that ligature of the œsophagus was not the innocuous operation which Orfila believed it to be; that in most cases it was followed by grave symptoms, and that when permanently applied, it was inevitably fatal. The bearing of these statements is clearly this:—Orfila left on record a large number of experiments in which he had placed various substances in the stomachs of dogs, and then tied the œsophagus. If, now, the later observers are right, and this operation is alone competent to destroy life, nearly all of Orfila's experiments will demand a new and more rigorous scrutiny.

The paper of MM. Bouley and Reynal was referred to a commission, composed of MM. Begin, Bouley, Jobert, Larrey, Rénault, and Trousseau, the latter of whom acted as reporter. To the same commission were also referred additional communications on the same subject by M. Orfila, a nephew of the toxicologist, Follin, Sédillot, Colin, Szumowski, and Jobert.

After a patient, experimental criticism of the views of these several observers, the commission arrived at the following conclusions:—

1. Ligature of the œsophagus causes symptoms of a serious nature, which should not be neglected in toxicological studies.

2. These symptoms are more or less grave as the ligature is more or less rightly applied.

3. Permanent ligature of this canal causes death in nine-tenths of the cases.

4. The maximum duration of life, under these circumstances, having been six days, doubt is cast upon the supposed toxicological characters of substances tested by ingestion and subsequent ligature of the œsophagus, when death follows their administration only after the lapse of two, three, four, five, or six days, and with still more reason when it is yet longer delayed.

5. The symptoms which follow the permanent application of a ligature to the œsophagus, are profound prostration, supervening at the close of twenty-four hours.

6. The consecutive lesions are inflammation of the nerves which accompany the œsophagus, with or without purulent formations in the track of the wound, so that where these complications attend the administration of poisons retained in the stomach by ligature of the œsophagus, their supposed effects are to be regarded with suspicion, owing to the impossibility of referring the accidents in question to the supposed poison, or to the means used to retain it in the intestinal cavities.

7. The temporary ligature of the canal is fatal but in three of one hundred cases.

8. It is hence inferred that when the ligature is to be used, it should be applied without great pressure, and should be removed within six hours.

In commenting upon the report here analyzed, M. Brown-Séquard states that two experiments are needed to make the matter complete—First, to irritate the nerves of the œsophagus without obstructing that canal; and second, to tie it after cutting the said nerves. To try the first of these, he divided the canal longitudinally, and fixed in it a tube by the aid of two ligatures, so that deglutition and vomiting were still possible. The accidents observed by MM. Bouley and Reynal showed themselves in this case, but to a less extent than when the canal was tied without the use of a tube.

In the second experiment, the two recurrent nerves were divided and a ligature placed about the canal near to the thorax. In this case, the phenomena recorded by Bouley and Reynal failed to appear! The symptoms observed by these gentlemen were therefore due in all probability to reflex impressions affecting the buccal, pharyngeal, salivary, bronchial, and gastric secretions, and analogous to the flow of tears which follows irritation of the cornea.—(*Journal de Physiologie*, October, 1858.)

VIII.—*On the Amyloid Corpuscles as Normal Productions on the Surface of the Skin.* By M. L. LUYS.

In October, 1853, Virchow pointed out to the *Académie des Sciences* of Paris, the existence of a particular substance in the human body, giving rise to the same chemical reactions as vegetable cellulose. This discovery was confirmed by Carter, of Edinburgh, (1855 and 1858,) and the result of the researches of these two observers was, that amyloid corpuscles were found in nearly all histological tissues. Virchow considered this substance a degeneration, a pathological condition. Carter, on the contrary, believed it to be a normal production, connected with the regular exercise of physiological phenomena. It is interesting to compare these investigations with the discoveries made by M. Cl. Bernard in regard to the sugar-forming substance, and to trace thus an additional resemblance between the phenomena which take place in the animal economy, and those which are observed in the vegetable organism.

In a memoir read before the *Société de Biologie*, M. Luys has endeavored to prove—1. That the skin is endowed, in its physiological and pathological condition, as all other tissues of the organism, with the property of producing amyloid corpuscles. Carter pointed out their existence only in certain cases of ichthyosis, in the deep layers of the dermis, and consequently believed that their presence in these cases was owing to a morbid state. 2. That these corpuscles present the reactions of amyloid substances.

The amyloid corpuscles are obtained by scraping the surface of the moistened skin with the back of a scalpel. They are more abundant on the nape of the neck, and on the scalp, than anywhere else. The epithelial lamellæ, which the comb or brush removes, are literally covered with a great quantity of these very corpuscles. The author has convinced himself, by careful experiments, that they came neither from the dust suspended in the atmosphere, nor from the starched linen worn on the body. The fact that amyloid corpuscles exist on the surface of the skin of the fœtus, at the moment when it leaves the uterine cavity, can be considered decisive. The corpuscles derived from this source have served to establish the typical characters.

The amyloid corpuscles are numerous; their dimensions vary from the hundredth to the four hundredth of a millimètre; transparent, pale, of disk-like form, resembling that of a ship biscuit, depressed in the centre, and appearing, consequently, slightly biconcave; homogeneous, of even fracture, and containing no cavity.

M. Luys has found them to possess the following chemical characters, which have been confirmed by the researches of M. Berthelot:—

They are insoluble in cold water, ether, in absolute alcohol, either cold or on heating, in spirits of turpentine, and in ammonia. After having been subjected to contact with these substances, they are always susceptible of being colored by iodine. In a solution of potassa or soda they swell, and become paler. Iodine gives them instantaneously a dark violet, nearly black color; on addition of sulphuric acid, the color changes to a clear blue.

If corpuscles thus darkened by iodine, are heated in a little water, they become paler as the heat of the liquid increases, and disappear entirely on ebullition; when the temperature afterwards decreases again, they reappear gradually with their primitive color. If corpuscles colored by iodine are subjected to the action of dry heat, they assume a Sienna-red color.

Although this substance, in the very small proportions in which it was obtained from the skin of the fœtus, has not yet given rise, in the hands of M. Luys, to the characteristic reactions of starchy matter, the formation of sugar, the author believes himself, nevertheless, authorized in saying that it approaches and presents the greatest analogies with vegetable starch.

The mode of formation, and the use of this amyloid substance, have not yet been ascertained, and demands further researches.—(*Gazette Médicale*, 1859, No. 1.)

